# Health workforce attrition in the public sector in Kenya: a look at the reasons

**DOI:** 10.1186/1478-4491-7-58

**Published:** 2009-07-21

**Authors:** Slavea Chankova, Stephen Muchiri, Gilbert Kombe

**Affiliations:** 1 International Health Division, Abt Associates Inc., Bethesda, Maryland, USA; 2 Ministry of State for Planning, National Development and Vision 2030, Nairobi, Kenya

## Abstract

**Background:**

Kenya, like many other countries in sub-Saharan Africa, has been affected by shortages of health workers in the public sector. Data on the rates and leading reasons for health workers attrition in the public sector are key in developing effective, evidence-based planning and policy on human resources for health.

**Methods:**

This study analysed data from a human resources health facility survey conducted in 2005 in 52 health centres and 22 public hospitals (including all provincial hospitals) across all eight provinces in Kenya. The study looked into the status of attrition rates and the proportion of attrition due to retirement, resignation or death among doctors, clinical officers, nurses and laboratory and pharmacy specialists in surveyed facilities.

**Results:**

Overall health workers attrition rates from 2004 to 2005 were similar across type of health facility: provincial hospitals lost on average 4% of their health workers, compared to 3% for district hospitals and 5% for health centres. However, there are differences in the patterns of attrition rates by cadre. Attrition among doctors and registered nurses was much higher at the provincial hospitals than at district hospitals or health centres, whereas the opposite pattern was observed for laboratory and pharmacy staff (lost at a higher rate in lower-level facilities). In provincial hospitals, doctors had higher attrition rates than clinical officers, and registered nurses had higher attrition rates than enrolled nurses. In contrast, attrition of enrolled and registered nurses in district hospitals and health centres was similar. The main reason for health worker attrition (all cadres combined) at each level of facility was retirement, followed by resignation and death. However, resignation drives attrition among doctors and clinical officers; retirement accounts for the main share of attrition among nurses and pharmacy staff; and death is the primary reason for attrition among laboratory staff, particularly in district hospitals. One limitation of the data is that sampling of health centres was non-random and the results may thus not be representative of all health centres.

**Conclusion:**

Our findings indicate that appropriate policies to retain staff in the public health sector may need to be tailored for different cadres and level of health facility. Further studies, perhaps employing qualitative research, need to investigate the importance of different factors in the decision of health workers to resign.

## Background

Human resources are the foundation of a health system and a key prerequisite to improving health outcomes [1]. Many countries in sub-Saharan Africa have endemic shortages of health workers, but the onset of the HIV/AIDS epidemic has worsened the problem by dramatically increasing the workload of hospital staff and directly affecting many health workers who have become infected with the virus [2-4].

In recent years, the situation of human resources for health (HRH) in many sub-Saharan African countries has been commonly described as "the crisis in human resources for health" [5-7]. A key contributor to the crisis is attrition of the health workforce, measured by the number of health workers who permanently leave their posts. Attrition is due to a number of reasons, including retirement, death, dismissal and voluntary resignation by health workers who leave the public health sector to work in the private sector, for more attractive occupations in the home country, or to emigrate to work in health facilities in richer countries, in search of better pay and working conditions. [8,9]

In Kenya, as in other countries in sub-Saharan Africa, the HRH crisis has become a major challenge for health service delivery and for achieving the health-related Millennium Development Goals [4,10]. The public health sector in Kenya provides about half of health care services in the country [11].

The government has recognized that the emergence and re-emergence of infectious diseases such as HIV/AIDS, TB and malaria have increased the demand for health services, putting an additional stress on the existing human resources in the public health sector [12]. Prevalence of HIV/AIDS in Kenya remains one of the highest in the region, at 7.4% in 2007 [13].

As Kenya started receiving support for HIV/AIDS, TB, malaria and immunization services from international donors such as PEPFAR, GFATM and GAVI in 2004 – 2005, the country's ability to translate such funding into improved and equitable health outcomes was threatened by the lack of sufficient human resources in the health sector: in the public sector there were three doctors and 49 nurses per 100 000 population (compared to a ratio of 143 nurses per 100 000 population recommended by the World Health Organization), and more than half of all health personnel and 80% of doctors were based in urban areas [14]. Shortages and misdistribution of HRH in the public sector may also pose a major challenge to Kenya in reaching the health-related Millennium Development Goals [15].

Recognizing this problem, the government of Kenya has declared shortages of health workers to be a major challenge to health development [16]; improving HRH has become a top priority. In 2005, the Ministry of Health conducted a human resource mapping and verification census of all public health facilities in Kenya, finding understaffed primary care facilities with relative overstaffing of hospitals and lower health worker-to-population ratios in poorer provinces [14].

Effective HRH planning and policy formulation in Kenya and elsewhere require sound empirical evidence on why and at what rate health workers leave the public health sector. However, while anecdotal evidence of high attrition rates among health workers in sub-Saharan African countries abounds, most countries have weak human resource information systems (HRIS) that cannot provide adequate data on the rates of health worker attrition.

One way to obtain empirical data on HRH attrition, when the data are not routinely available from a HRIS, is a health facility survey. Surveys of available HRH resources in developing countries have become more prevalent in recent years, often as part of larger health services provision surveys [17]. However, service provision surveys focus on the numbers and training characteristics of health workers, and do not include questions about HRH attrition. A number of recent studies focusing specifically on HRH have documented the rate and main reasons of HRH attrition in Ethiopia [18], Zambia [19], and Nigeria [20], whereas other studies have explored the reasons why health workers leave, or intend to leave, their posts [3,21,22].

In this article, we report the findings on HRH attrition from a nationwide health facility survey in the public health sector in Kenya. The survey documented the overall rate and reasons for attrition among key cadres of health workers, including doctors, clinical officers, nurses and laboratory and pharmacy specialists. The empirical evidence we present illustrates differences in attrition patterns by level of health facility. Our study aims to add to the evidence on HRH attrition in sub-Saharan Africa, to provide evidence for HRH planning in Kenya and to lay the groundwork for further research needed to support HRH policy decisions in Kenya and beyond.

## Methods

The results presented here are based on data collected as part of a health facility survey conducted in Kenya in 2005 by the USAID-sponsored Partners for Health Reform *plus *project and the Kenya Ministry of Health. The survey covered 74 primary and secondary public sector health facilities in all provinces of the country (Table 1). All seven provincial general hospitals were included in the sample. A sample of 23 districts was selected across the eight provinces. In each selected district, the district hospital (if there was one) was included in the sample, and up to three health centres were selected.

**Table 1:** Number of health facilities in study sample^a^

**Province**	**Provincial hospitals**	**District hospitals**	**Health centres**
Central	1 (1)	3 (15)	5 (66)

Coast	1 (1)	3 (16)	9 (38)

Eastern	1 (1)	2 (28)	5 (70)

Nairobi	- (0)	1 (4)	6 (10)

North Eastern	1 (1)	- (4)	2 (13)

Nyanza	1 (1)	2 (26)	8 (94)

Rift Valley	1 (1)	2 (38)	8 (132)

Western	1 (1)	2 (13)	9 (66)

Total	7 (7)	15 (144)	52 (489)

^a ^Total number of public health facilities in sampling frame shown in brackets.

Selection of health centres was not random but was based on their geographical proximity to the district hospital, with selection preference for health centres that provided HIV/AIDS or TB services (reflecting additional study objectives related to provision of such services). Therefore it is possible that the health centres in our sample are different from the average health centre in their area (e.g. they may be larger in size, better equipped or situated in a larger town or village). As a result, health centres in our sample may experience different patterns of health worker attrition, compared to the average for health centres in their district. Our study does not include dispensaries, the lowest health facility level.

A health facility questionnaire was administered to cognizant staff members, including the medical director or the staff in charge of HR management or administration. Data were collected directly from facility registers; additional information was provided by those interviewed. The questionnaire collected data on the number of health workers employed in 2004 and 2005, as well as on the number who had left and the number who had joined the facility between mid-2004 and mid-2005, including the reason for leaving. The list of reasons included resignation, retirement, death and transfer to another health facility within the public sector. The data collection took place in October – November 2005. EpiInfo data screens were used for the data entry; all analysis was performed using Intercooled Stata v.8.0 software.

We calculated average attrition rates by type of facility for selected several cadres of health workers (excluding foreign workers). In the results presentation, we group laboratory technicians and laboratory technologists in a category that we call "laboratory staff", and we group pharmacists and pharmaceutical technologists in a category that we call "pharmacy staff". Attrition rate for each facility was computed as the number of health workers who left the facility between mid-2004 and mid-2005, divided by the number of health workers who were employed by the facility in mid-2004. We then computed an average of the attrition rates for all facilities of a given type (e.g. health centres) by health worker category. In addition, we computed the share of total attrition at a health facility that was due to resignation, retirement or death, and compared the distribution of attrition reasons across types of health facilities and health worker cadres.

It is important to keep in mind that all attrition rates presented in this study were computed at the individual facility level, and then averaged across facilities. Accordingly, the attrition results reflect the situation faced by the average facility. One limitation of our study is that some results are based on small sample sizes (e.g. if only a few facilities report attrition of a given cadre, then the analyses of reasons for attrition are based on data from only these few facilities).

As the purpose of this study was to inform policy-makers on the rate at which health workers leave public health facilities and the reasons for that in Kenya, our calculation of attrition took into account only those who had permanently left the public health sector (i.e. those who resigned, retired or died).

## Results

Table 2 summarizes the number of health workers in the health facilities included in our sample. At each level of health facility, the largest category of health workers was enrolled nurses, followed by registered nurses, clinical officers, doctors and pharmacy and laboratory specialists.

**Table 2:** Number of health workers per facility in study sample^a^

	**Provincial general hospitals** **(n = 7)**	**District hospitals** **(n = 15)**	**Health centres** **(n = 52)**
Doctors	38 (12–72)	15 (5–53)	-

Clinical officers	40 (17–108)	19 (10–29)	1.1 (0–3)

Registered nurses	89 (28–218)	34 (9–72)	1.3 (0–4)

Enrolled nurses	173 (6–322)	107 (46–212)	5.8 (0–16)

Laboratory technicians and technologists	11.1 (4–33)	4.3(1–8)	0.2 (0–2)

Pharmacists and pharmacy technicians	21.6 (10–37)	12.7 (7–20)	1.0 (0–4)

^a ^Range shown in brackets.

While the average number of enrolled nurses was substantially higher than that of registered nurses, the number of clinical officers in district and provincial general hospitals was about the same as the number of doctors in these facilities. The average provincial general hospital had 38 doctors, 40 clinical officers, 262 nurses, 11 laboratory specialists and 22 pharmacy specialists. The average district hospital in our sample had about half this number of health workers (15 doctors, 19 clinical officers, 141 nurses, 4 laboratory and 13 pharmacy specialists). In health centres, there was on average one clinical officer, seven nurses (most of them enrolled nurses) and one pharmacy specialist. One in five health centres had a laboratory specialist.

### Overall attrition rates

**Figure 1:**
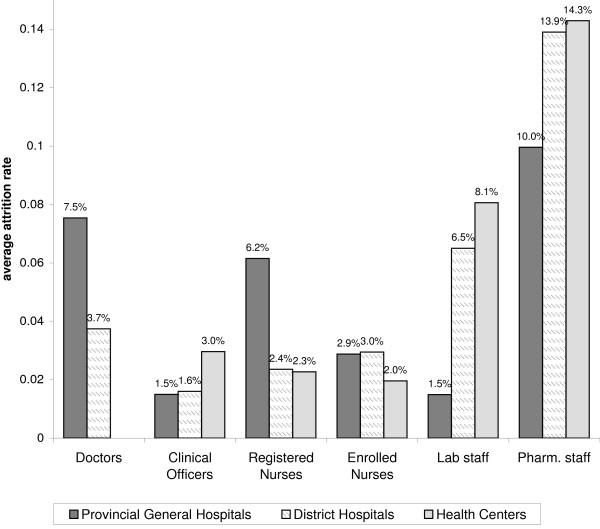
**Average attrition rate by HRH category and type of health facility**.

The attrition rate for the total number of health workers (all cadres included in this study combined) was not substantially different by type of health facility: provincial hospitals lost on average 4% of their health workers, compared to 3% for district hospitals and 5% for health centres. However, there were marked differences in the patterns of attrition rates by HRH cadre (Figure 1).

At all levels of care, attrition was highest among pharmacy staff. Comparison of attrition rates by type of facility for each cadre showed some interesting patterns. Attrition among doctors and registered nurses was much higher at the provincial hospitals than at district hospitals or health centres, whereas the opposite pattern was observed for laboratory and pharmacy staff (lost at a higher rate in lower-level facilities).

On average, provincial hospitals lost doctors at twice the rate in district hospitals (8% and 4%, respectively), while registered nurses at provincial hospitals were lost at three times the rate of registered nurses in district hospitals (6% and 2%, respectively). On the other hand, the number of laboratory staff decreased by less than 2% in provincial hospitals, but by 7% to 8% in district hospitals and health centres. While attrition for pharmacy staff was on average 10% in provincial hospitals, it reached 14% in district hospitals and the few health centres that had pharmacy staff (only 13% of health centres had any pharmacy staff).

At all types of facilities, attrition among clinical officers was substantially lower than for doctors: about 2% at provincial hospitals and district hospitals and 3% at health centres. While the attrition rate for registered nurses was twice as high as for enrolled nurses at provincial hospitals (6% and 3%, respectively), attrition for these two cadres at lower levels of care was about the same (2% to 3% in district hospitals and health centres).

### Distribution of reasons for attrition across cadres and health facility type

**Figure 2:**
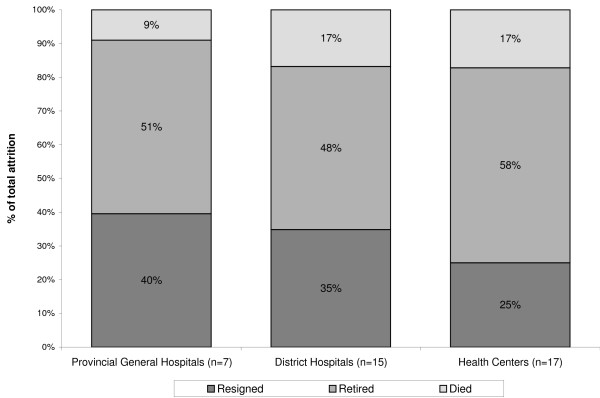
**Distribution of reasons for HRH attrition by type of health facility**.

The main reason for health worker attrition at each level of facility, when looking at all cadres combined, was retirement (accounting for 48% to 58% of total attrition at the average facility), followed by resignation and death (Figure 2). Resignation accounted on average for 40% of HRH attrition in provincial hospitals, 35% of attrition in district hospitals and 25% of attrition in health centres.

**Figure 3:**
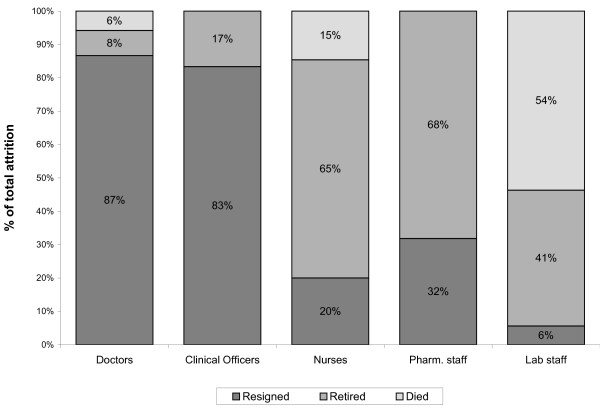
**Reasons for attrition of health workers in provincial and district hospitals**.

However, a look at the reasons for attrition by type of health workers shows different patterns. First, we looked at hospitals, which have each of the health worker cadres included in the study. We combined provincial and district hospitals for the purposes of this analysis. In hospitals, resignation was the leading reason for loss of doctors and clinical officers (accounting for more than 80% of attrition), while the leading cause of attrition among nurses and pharmacy staff was retirement, accounting for two thirds or more of attrition in these groups (Figure 3). The leading reason for attrition among laboratory staff in hospitals was death (54% of attrition in hospitals that reported loss of laboratory staff), while resignation accounted only for 6% of attrition.

The pattern of attrition reasons for each cadre was the same at provincial hospitals and district hospitals, except for registered nurses and laboratory staff. Resignation accounted for about half of attrition in provincial hospitals, but for only 17% in district hospitals (where retirement was the leading reason for loss of registered nurses). While resignation was the only reason for laboratory staff lost in provincial hospitals, death was the leading reason for laboratory staff attrition in district hospitals, accounting for 75% of attrition of laboratory staff.

**Figure 4:**
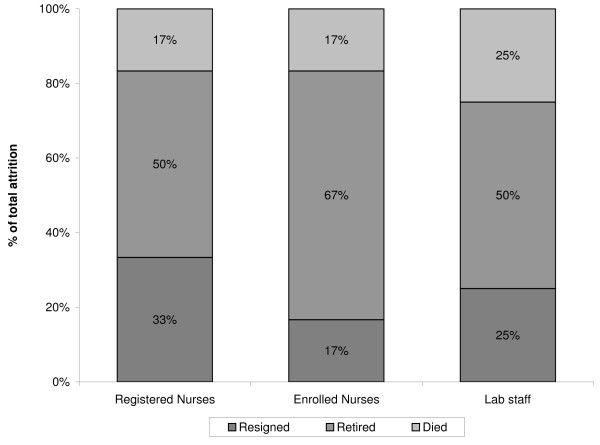
**Reasons for attrition of health workers in health centres**.

Unlike hospitals, the proportion of health centres in our sample that reported loss of health workers for each cadre was very low. Between two and six of the 52 health centres reported loss of health workers from a given cadre, although nearly all health centres had at least one enrolled nurse (92%), 85% had at least one registered nurse, 87% had at least one clinical officer and 60% had laboratory staff. In health centres, retirement was the leading reason for attrition among all cadres combined, accounting for at least half of attrition (Figure 2). Retirement remained the leading reason for attrition when looking separately at registered nurses, enrolled nurses and laboratory staff (the cadres that are most prevalent in health centres) (Figure 4).

Resignation was the reason for 33% of attrition among registered nurses, 17% among enrolled nurses and 25% among laboratory staff. Only two of the 45 health centres with clinical officers reported loss of this cadre, with half of attrition due to retirement and the rest due to resignation. Similarly, only two of the seven health centres that had pharmacy staff reported loss of such staff, all due to retirement.

## Discussion

Our results highlight several areas for discussion. First, the overall attrition rate (all health worker cadres combined) at the average provincial hospital was not much different from attrition at the average district hospital or health centre in our sample. This finding is in contrast with the typical concern voiced by researchers and policy-makers that primary health facilities, in sub-Saharan Africa and elsewhere, tend to lose health workers at a higher rate, compared to secondary and tertiary facilities.

In hospitals, doctors had much higher rates of attrition, compared to clinical officers, although resignation was the predominant reason for attrition in both cadres. This finding may reflect a recent trend for doctors, who may be moving completely away from public service rather than staying on with the dual employment opportunity (often referred to as "moonlighting") that has been on the books for years. The differential rates of attrition between doctors and clinical officers may thus reflect that doctors are more likely to emigrate for work in health facilities abroad or to go completely into private practice or employment in the NGO sector in the home country (which are not opportunities as readily available to clinical officers).

Attrition among registered nurses in provincial hospitals was, on average, twice as high as the rate of attrition of enrolled nurses. While resignation accounted for about half of attrition among registered nurses at this level, the loss of enrolled nurses was nearly all due to retirement. By contrast, at lower facility levels, registered and enrolled nurses had similar rates of attrition, mostly explained by retirement. This may reflect the higher international mobility and more numerous alternative employment opportunities available to registered nurses (in comparison with enrolled nurses), particularly in urban areas where the provincial hospitals are located.

The high levels of attrition among pharmacy staff across all facility levels (10% to 14%) were due primarily to retirement. This may have been a result of non-replacement of this cadre over time, leading to an aged pool of these personnel. That resignation was not as prominent a reason for attrition in this group may reflect the lack of better opportunities in the private health sector for pharmacy specialists.

Attrition among laboratory staff at provincial hospitals and health centres was explained by retirement, while the predominant reason in district hospitals was death. The high proportion of deaths accounting for attrition among laboratory staff in district hospitals is worrying and must be explored further to identify the causes. That retirement also accounts for a high proportion of laboratory staff attrition indicates that this cadre may be an elderly section of the workforce and that the provision of laboratory services may need to be addressed urgently.

There are several areas in need of in-depth future research, based on these results. Our finding that resignation was the predominant reason for relatively high attrition among doctors and registered nurses in provincial hospitals can benefit from further research on the factors that led these cadres to resign. Similarly, research among the cadres where resignation accounted for a small share of health worker loss (such as enrolled nurses and laboratory and pharmacy staff) may shed light on factors successful in keeping these health workers at their posts. Qualitative research methods can be particularly relevant in investigating such factors. This type of further research will inform retention policies and help prioritize resources towards areas that are most important for keeping the different health cadres in their posts – whether higher salaries, professional opportunities or other factors.

Studies from other countries as to why health workers resign have found that the main reasons are low pay; poor working and living conditions at the sites where they are posted [1,23]; and reasons related to the HIV/AIDS epidemic, such as fear of becoming infected on the job and overwhelming workload and stress induced by caring for, and seeing high death rates among, HIV/AIDS patients [4]. For health workers in rural areas, an additional problem is inadequate quality of housing, transport and schools for their children. Those "push" factors combine with "pull" factors such as better pay and opportunities available in other occupations or health facilities abroad [21,22,24-27]. Investigating the role of these factors for health worker resignations in Kenya – particularly whether different factors play a role for different cadres – would inform and strengthen retention policies in Kenya.

A few limitations of our study need to be highlighted. First, our survey covered only one year, and it is possible that attrition trends may vary over years. Second, our survey did not measure absenteeism among health workers, which is another aspect of the shortage of health workers at facilities [4]. While further research and interventions to address the attrition of health workers from the public sector are of key importance, absenteeism among health workers would also need to be on the research and policy agenda. Lastly, this study did not measure attrition differences between rural and urban areas, or attrition and reasons for resignation by gender, which are important areas where further research would be particularly valuable.

## Conclusion

The evidence provided in this study highlights the need to develop appropriate policies to retain staff in the public health sector that may need to be tailored for different cadres and level of health facility. Although there has been heavy investment by both the Government and the development partners in the health sector, it is now evident that without earmarking some funds to increase the pool of human resources, Kenya is unlikely to achieve the health related MDGs.

In the last few years, development partners have provided funds to hire additional workers on contract; those workers are posted to districts on the condition that they must remain in the posted station for the entire duration of the contract. This has assisted in retaining staff, especially in underserved areas. Further research into differences in attrition patterns by gender or region would help in designing retention incentives and shaping the composition of intakes to medical and nursing schools.

One solution to alleviating shortages of doctors that is gaining prominence in the HRH debate and practice is increasing the numbers of non-physician clinicians (such as clinical officers) and shifting tasks that can be handled by non-physicians [28,29]. Other policy suggestions to address resignations are to improve the salary package for health workers and the working environment in public health facilities. As the HRH crisis persists, it is ironic that some developing countries are considering abolishing the training of enrolled nurses – who are more likely to stay at their posts in the public sector, compared to registered nurses – in favour of more expensive and qualified registered nurses.

## List of abbreviations

GAVI: Global Alliance for Vaccines and Immunization; GFATM: Global Fund to Fight AIDS, Tuberculosis and Malaria; PEPFAR: President's Emergency Plan for AIDS Relief; USAID: United States Agency for International Development

## Competing interests

The authors declare that they have no competing interests.

## Authors' contributions

SC analysed the data and drafted the manuscript. GK and SM led the design of the study, managed the data collection and contributed to the manuscript draft. All authors read and approved the final manuscript.
